# Neuron-Derived Exosome Proteins May Contribute to Progression From Repetitive Mild Traumatic Brain Injuries to Chronic Traumatic Encephalopathy

**DOI:** 10.3389/fnins.2019.00452

**Published:** 2019-05-08

**Authors:** Edward J. Goetzl, Aurélie Ledreux, Ann-Charlotte Granholm, Fanny M. Elahi, Laura Goetzl, Jade Hiramoto, Dimitrios Kapogiannis

**Affiliations:** ^1^Department of Medicine, University of California, San Francisco, San Francisco, CA, United States; ^2^Knoebel Institute for Healthy Aging, University of Denver, Denver, CO, United States; ^3^Department of Neurology, Memory and Aging Center, University of California, San Francisco, San Francisco, CA, United States; ^4^Department of Obstetrics, Gynecology and Reproductive Sciences, Health Sciences Center at Houston, University of Texas, Houston, TX, United States; ^5^Division of Vascular Surgery, Department of Surgery, University of California, San Francisco, San Francisco, CA, United States; ^6^Laboratory of Neurosciences, Intramural Research Program, National Institute on Aging, Baltimore, MD, United States

**Keywords:** amyloid, cellular prion protein, phospho-tau, synaptogyrin-3, Alzheimer disease

## Abstract

The recent recognition that Alzheimer disease-like pathology may be found in chronic traumatic encephalopathy (CTE) even after acute mild traumatic brain injury (mTBI) has increased the urgency of elucidating mechanisms, identifying biomarkers predictive of high risk of development of CTE, and establishing biomarker profiles indicative of impactful effects of treatments. Of the many proteins that are loaded into neuron-derived exosomes (NDEs) from damaged neurons after acute TBI, the levels of prion cellular protein (PRPc), coagulation factor XIII (XIIIa), synaptogyrin-3, IL-6, and aquaporins remain elevated for months. Prolonged heightened expression of aquaporins and IL-6 may account for the persistent central nervous system edema and inflammation of CTE. PRPc, XIIIa and synaptogyrin-3 bind and concentrate neurotoxic forms of oligomeric amyloid β peptides or P-tau for delivery into neurons at or distant from the site of trauma. Our progression factor hypothesis of CTE asserts that physiological neuronal proteins, such as PRPc, XIIIa, synaptogyrin-3, IL-6 and aquaporins, that increase in concentration in neurons and NDEs for months after acute TBI, are etiological contributors to CTE by either direct actions or by recruiting neurotoxic forms of Aβ peptides or P-tau. Such progression factors also may be useful new targets for development of drugs to prevent CTE.

## Introduction

Traumatic brain injury (TBI) is the term used to describe mechanical injury to the parenchymal tissues and meninges of the brain, and the subsequent series of further damaging proteolytic, oxidative, inflammatory, and proteinopathic responses (Langlois et al., 2006; Roozenbeek et al., 2013; Quillinan et al., 2016). Several million TBIs occur annually in the United States, of which approximately 80% are defined as mild traumatic brain injury (mTBI) (Faul and Coronado, 2015; Taylor et al., 2017). A high frequency of repetitive mTBIs is observed in young adults from participation in sports and from the shock waves created by military explosives (Broglio et al., 2009; Champion et al., 2009; Wilk et al., 2012; Papa et al., 2015; McKee et al., 2016). It is clear that moderate and severe TBI predispose to both more prevalent and earlier onset Alzheimer disease, and to CTE (Barnes et al., 2014; Gardner et al., 2014; McKee et al., 2016; Perry et al., 2016). Despite the far higher prevalence of mTBI than moderate and severe TBI, it has only recently been recognized that repetitive mTBIs also increase the risk of subsequent development of CTE and dementia (Crane et al., 2016; Barnes et al., 2018). Our understanding of the mechanisms of acute mTBI resulting from a single concussive blow and CTE following repeated episodes of acute mTBI is incomplete, in part due to the past lack of sensitive and accurate biomarkers for the many constituent abnormalities.

The complex matrix of pathways interacting in the course of an mTBI begin with disruption of the blood-brain barrier (BBB) and axonal injury. BBB damage is manifested maximally within hours by morphological changes in endothelial cells, pericytes and astrocytes, increases in the number of perivascular monocytes, and elevated blood levels of tight-junction and astrocyte-specific proteins (Blyth et al., 2009; Higashida et al., 2011; Obermeier et al., 2013; Kuriakose et al., 2018). Subsequent edema of neurons and interstitial tissues follows with increased brain tissue levels of proteins from damaged neuronal framework structures, channels, transporters, and vesicle transport systems (Hue et al., 2016; Kuriakose et al., 2018). Inflammation develops in the same time period of initial responses to acute mTBI, as shown by increased brain tissue levels of cytokines, such as IFN-α, IL-1β, IL-6, and MCP, oxygen radicals and matrix metalloproteinases (Helmy et al., 2011; Woodcock and Morganti-Kossmann, 2013; Yan et al., 2014; Gyoneva and Ransohoff, 2015; Lozano et al., 2015; Simon et al., 2017; Roselli et al., 2018). This period also is characterized by increased expression of stress response and pro-apoptotic factor genes (Mettang et al., 2018).

Onset of more persistent features of CTE months or years after a TBI may be foreshadowed by sustained neuronal elevations of elements of proteinopathic neurodegeneration and chronic inflammation (DeKosky et al., 2013). Although neuropathogenic bridges linking acute mTBI to CTE are poorly understood, some mechanisms have been recognized. Within 24 h of acute TBI in rats, hippocampal and cortical brain tissues showed transient increases in beta-secretase-1 (BACE-1) (Blasko et al., 2004). In humans suffering single-incident severe TBI, increases in brain tissue levels of APP and Aβ peptides as well as diffuse Aβ plaques may be found within hours and persist variably (DeKosky et al., 2013). In contrast, the predominant early neuropathology in CTE following repetitive mTBIs is patchy distribution of neuropil threads and neurofibrillary tangles of P-tau throughout the neocortex (DeKosky et al., 2013). Plasma levels of tau-positive exosomes were abnormally elevated in former professional football players, exposed to repetitive mTBIs, and these increases correlated with decreases in memory and psychomotor speed (Stern et al., 2016). Although many abnormalities of cognition and affect or mood are similar in subjects suffering from CTE after acute severe TBI or repetitive mTBI, relationships between these neurocognitive abnormalities and differing proteinopathic etiologic mechanisms have not been delineated (Prince and Bruhns, 2017; Roy et al., 2017; Dailey et al., 2018).

Investigations of a wide range of neurally derived proteins and of systemic predominantly lipid metabolites in CSF and plasma have not yielded biomarkers that reliably assess the severity of mTBI, predict progression of mTBI to CTE, or offer useful targets for therapy (DeKosky et al., 2013; Agoston et al., 2017; Werhane et al., 2017; Kim et al., 2018). Changes in blood levels of some individual proteins, such as tau, s100B, neurofilament light chain, ubiquitin protein hydrolase-1 and neuron-specific enolase, showed moderate diagnostic and/or prognostic value in some circumstances, but had limited neural specificity, late expression and lack of cross-correlation (Di Battista et al., 2013). A comprehensive gas chromatographic analysis of sera obtained within 2 weeks of mTBI or more severe TBI showed elevations of two medium-chain fatty acids and 2,3-bisphosphoglyceric acid, that were significantly associated with the severity of TBI and added value to clinical and radiological imaging results (Oresic et al., 2016). Six plasma metabolites, including two fatty acids, three phospholipids and tauroursodeoxycholic acid, that were quantified at <6 h to 7 days after mTBI, significantly distinguished mTBI athletes from matched uninjured teammate controls throughout the week after mTBI (Fiandaca et al., 2018). However, as for the prior investigation of metabolomic lipids, these analytes have no known specific CNS functions and statistical characteristics of data for two independent cohorts showed major differences. It thus seems appropriate now to investigate subjects with acute or chronic repetitive mTBI by quantifying alterations in levels of their endosomal and other membrane CNS proteins, that are accessible in the protected cargo of plasma NDEs. The multiphasic and multicomponent set of events characteristic of all TBIs has suggested the importance of selecting biomarkers that probe both intracellular processes and diverse relevant mechanisms.

## Plasma Neuron-Derived Exosome Protein Abnormalities in mTBI

Application of established technology to enrich the NDE subset of extracellular vesicles in plasma has permitted initial elucidation of the changes in exosome trafficking and levels of cargo proteins characteristic of mTBIs. A study of military personnel suffering mTBI and subsequent chronic unspecific CNS symptoms revealed significant increases in CD81 exosome marker-normalized NDE levels of total tau, Aβ42, and IL-10 relative to deployed controls without a history of mTBI (Gill et al., 2018). Although the precise timing of episodes of mTBI was not determinable, most had been in combat at least once during the year preceding testing. Significant associations were identified between post-concussive symptoms and increases in NDE tau and between symptoms of post-traumatic stress disorder and increases in NDE IL-10 (Gill et al., 2018).

A recent investigation of plasma levels of NDEs and a broader profile of NDE cargo proteins has been conducted in college athletes aged 18–26 years, who provided plasma within 1 week after a sports-related mTBI (acute mTBI, *n* = 18) or 3 months or longer after two to four mTBIs (chronic mTBI, *n* = 14) (Goetzl et al., 2019). Plasma levels of NDEs, assessed by closely correlated counts and levels of extracted CD81 exosome marker, were significantly depressed by a mean of 45% in acute mTBI (*P* < 0.0001), as compared to teammates without TBI (*n* = 21), but not in chronic mTBI. Mean CD81-normalized NDE concentrations of a range of functional brain proteins were significantly abnormal relative to those of uninjured teammate controls in acute but not chronic mTBI groups, including ras-related small GTPase 10 (Rab 10), 73% decrease; annexin VII, 8.8-fold increase; UCH-L1, 2.5-fold increase; αII spectrin fragments, 1.9-fold increase; claudin-5, 2.7-fold increase; and sodium-potassium-chloride cotransporter-1 (NKCC-1), 2.8-fold increase (all acute increases were significantly different from controls, *P* < 0.0001). Three physiological neural proteins whose NDE levels changed significantly in acute mTBI also maintained lesser alterations in chronic TBI. Aquaporin 4 showed 8.9-fold (*P* < 0.0001) and 3.6-fold (*P* = 0.0004) increases, and synaptogyrin-3 showed 3.1-fold (*P* < 0.0001) and 1.3-fold (*P* = 0.041) increases, whereas ras-related small GTPase 35 (Rab 35) exhibited 56% (*P* < 0.0001) and 33% (*P* = 0.0045) decreases, respectively, in acute and chronic mTBI. In chronic mTBI, there were elevated CD81-normalized NDE levels of usually neuropathologic β-amyloid peptide 1–42 (Aβ42), 1.6-fold (*P* < 0.0001 relative to controls); XIIIa, 1.3-fold (*P* = 0.042); P-T181-tau, 2.2-fold (*P* < 0.0001); P-S396-tau, 1.6-fold (*P* < 0.01); IL-6, 16-fold (*P* < 0.0001); and prion cellular protein (PRPc), 5.1-fold (*P* < 0.0001), with lesser or greater (XIIIa, IL-6, PRPc) increases in acute mTBI.

**FIGURE 1:**
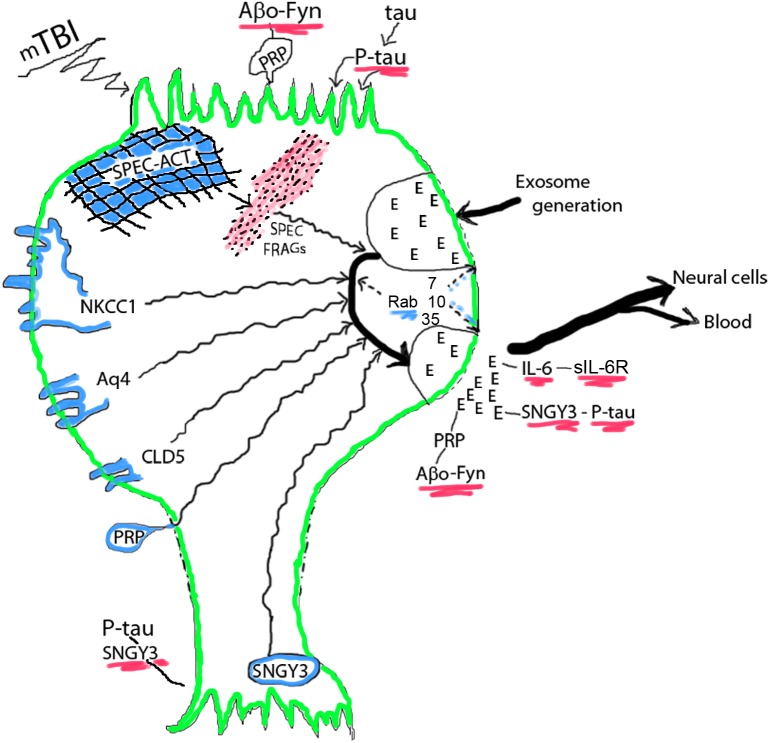
Acute mTBI-evoked alterations in NDEs. NDEs are generated at the plasma membrane surface of the cell body, loaded with diverse neuronal proteins in their endosomal course in multivesicular bodies, and secreted through secretory type multivesicular bodies. Cargo proteins are from membrane structures or the spectrin (SPEC)-actin (ACT) matrix and their levels in NDEs increase after mTBI. The small GTPases Rab 7, 10, 35, and others regulate the processes of NDE generation, trafficking and secretion, that all diminish after mTBI reduces Rab levels. Progression factors bind, concentrate and deliver neurotoxic proteins: oligomeric Aβ peptides (Aβo) by PRPc in a trimolecular complex with Fyn kinase at dendritic surfaces and in NDEs, P-tau by synaptogyrin-3 (SNGY3) at axonal and synaptic surfaces and in NDEs, and IL-6 by soluble(s)IL-6Rs. E, exosome. Color depictions are green for neuronal boundary, blue for normal cellular proteins that are found in NDE cargo and red for disrupted neuronal matrix proteins and neurotoxic progression factor complexes.

The acute decreases in Rab 10 and Rab 35 levels in NDEs, that are presumed to reflect neuronal levels of these small GTPases involved in vesicular formation, trafficking and secretion, may account for the observed acute decreases in plasma levels of NDEs, as discussed in the next section (Figure 1). The sustained decrease in NDE level of Rab35 distinguishes this response from the transient acute decrease in NDE level of Rab10. Levels of other NDE cargo proteins, that represent disposal of damaged neuronal proteins, instead significantly increased after acute mTBI but these increases were not all sustained in chronic mTBI. Of the acute mTBI NDE cargo responders, annexin VII has roles in neuronal survival and membrane transport functions, UCH-L1 targets damaged proteins by ubiquitination for elimination, αII spectrin with actin constitutes the neuronal periodic skeleton, claudin-5 is a component of the tight junction of the BBB, and NKCC-1 and aquaporins, respectively control ion and water movements required for normal neuronal functions (Creutz, 1992; Ohara et al., 1998; Brophy et al., 2011; Scharfman and Binder, 2013; Morita et al., 2014). In contrast, the NDE proteins that increased in concentration after chronic repetitive mTBIs without (only P-S396-tau) or with significant increases in acute mTBI are regarded predominantly as mediators of proteinopathic neurodegeneration, including total tau, P-T181-tau, P-S396-tau, Aβ42, IL-6, PRPc, and XIIIa. Of this group, PRPc and XIIIa, as well as the acute responder synaptogyrin-3 remain elevated through repetitive mTBIs and bind, concentrate and deliver the mediators of proteinopathic neurodegeneration P-tau or Aβ42 to other neurons.

Hypoxic or ischemic CNS damage in two disease states evoked some of the same changes in plasma levels of NDEs and/or cargo proteins as were observed in mTBI. In the first example, ten patients undergoing multi-branched endovascular repair of aortic aneurysms were studied pre-operatively, immediately postoperatively and the day after surgery. This form of surgery is often associated with ischemic hyperglycemic spinal damage and prolonged lower extremity weakness despite application of intra-operative counter-measures (Hiramoto et al., 2017). The mean level of NDEs fell significantly after surgery and returned the following day to that observed pre-operatively. The NDE mean level of Rab 10 also decreased post-operatively and rose to near normal the next day. NDE mean levels of PRPc, neurofilament light chain, NKCC-1, aquaporins and several synaptic proteins increased significantly after surgery and decreased to near normal the next day. This contrasts with the sustained elevations of PRPc, aquaporins and synaptogyrin-3 after mTBI. NDE levels of differently phosphorylated forms of insulin receptor signaling protein, changed post-operatively in accordance with development of insulin resistance. The mean NDE level of several other proteins, such as IL-6, rose post-operatively, and fell the next day, but remained significantly higher than that seen pre-operatively, which again raised the possibility of a subset of neural protein progression factors that might mediate subacute or chronic injury after hypoxia or ischemia.

The second example is neonatal hypoxemic-ischemic encephalopathy and early identification of affected infants that are not responding to standard therapeutic hypothermia. Neonatal NDEs in plasma obtained at 8, 10 and 14 h after initiation of hypothermia were enriched by absorption with anti-Contactin-2 antibody (Goetzl et al., 2018). CD81-normalized levels of neonatal NDE synaptopodin, synaptophysin, neuron-specific enolase and mitochondrial cytochrome C oxidase were quantified immunochemically. The most promising marker for all clinical outcomes was NDE synaptopodin, as the slope of change in its NDE level between 8 and 14 h of hypothermia was associated with clinically significant outcomes including persistent seizure activity requiring treatment (*p* = 0.02) and length of hospital stay (*P* < 0.001).

## Rab Small GTPases and NDE Trafficking in mTBIs

Rab small GTPases, the largest branch of the Ras superfamily, are highly abundant, and diverse organizers of intracellular membrane trafficking in the CNS (Kiral et al., 2018). The state of membrane association and activity of Rab GTPases are highly dependent on controlling interactions with numerous classes of accessory proteins. In the CNS, most Rab GTPases have multiple functions. For example, Rab 7 in neurons is a critical regulator of multivesicular body maturation from early endosomes, fusion of multivesicular bodies with lysosomes to form autolysosomes, and retrograde axonal transport of vesicles with the motor protein dynein into neuron cell bodies. Similarly, Rab 35 is important for the rejuvenation of synaptic vesicles from sorting/recycling endosomes and influences neuronal actin dynamics, largely by activating Rho GTPases (Chua et al., 2010). In contrast to the increases in some neuronal Rab GTPases observed in many neurodegenerative diseases (Kiral et al., 2018), NDE levels of Rab 7, Rab 10, and Rab 35 decrease in acute mTBI. Any NDE-related functional significance of decreased levels of some Rab GTPases in acute mTBI remains to be established.

## Potential NDE Protein Progression Factors in the Continuum from Repetitive mTBIs to Chronic Traumatic Encephalopathy (CTE)

It is too early in the course of elucidating acute mTBI-initiated pathways of pathogenesis of CTE for meaningful speculation about critical mechanisms. Further, we have no data for contributions of NDE miRNAs or lipids, or of astrocyte- or microglia-derived exosomes. However, a tentative analytical integration of existing data has identified several proteins in NDEs that may serve as factors mediating progression from repetitive mTBIs to CTE. Neuronal proteins that are elevated in NDEs acutely after mTBIs and remain significantly increased for 3 months or longer at levels higher than seen acutely include the directly neurotoxic Aβ42 and P-T181-tau (Goetzl et al., 2019). Neuronal proteins that are elevated in NDEs acutely after mTBIs and remain significantly increased for 3 months or longer at levels lower than seen acutely are aquaporins, synaptogyrin-3 and XIIIa, which lack direct neurotoxicity, as well as IL-6 and PRPc, that may diminish neuronal viability (Goetzl et al., 2019). Each of these latter mTBI-generated proteins is a candidate progression factor that promotes sustained neurotoxicity indirectly through enhancement of Aβ42 or P-tau effects or by other mechanisms.

**FIGURE 2:**
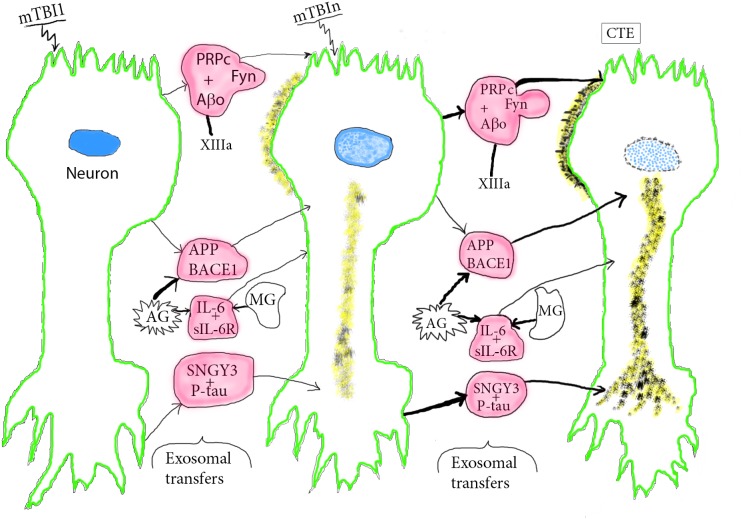
Course of pathogenesis of CTE by repetitive mTBIs. An initial mTBI leads to neuronal production of NDEs containing pathogenic complexes composed of PRPc+Aβo+Fyn, SNGY3+P-tau, and IL-6-sIL-6R that begin to damage the donor neurons at sites of mTBI and other neurons that receive the neurotoxic NDEs. Microglia (MG) and astrocytes (AG) produce MDEs and ADEs, respectively, that carry increased levels of APP, BACE-1, and IL-6 capable of further neurotoxic damage of neurons that receive these other sets of exosomes. This series of processes increases with subsequent episodes of mTBI and is enhanced by other progression factors, such as XIIIa that promotes oligomerization of Aβ to Aβo. P-tau neuropil fibers and Aβo deposits increase in size and density with repetitive mTBIs and lead to neuronal apoptosis. Color depictions are green for neuronal boundary, blue for the degenerating nucleus, yellow for Aβ, P-tau deposits, and red for NDEs with progression factor complexes and AG/MG products.

In the model proposed, mTBI damages and releases from neuronal membranes and other structures a wide range of functional proteins that are loaded into NDEs in endosomal pathways (Figure 1). No relationships have yet been established between the severity of a single mTBI and the magnitude or duration of increases in NDE levels of the proteins being removed by the exosomal pathway, but indirect evidence suggests that most such increases last for less than 3 months after a single mTBI. Transient decreases in NDE levels and presumptively neuronal levels of Rab 7, 10, and 35 reduce formation and secretion of NDEs for days after an acute mTBI with potential back-up of released proteins in the neurons. mTBI increases NDE and neuronal levels of the binding partners PRPc with Aβ peptides, especially oligomeric forms of Aβ (Aβo), synaptogyrin-3 with P-tau species, and IL-6 with soluble IL-6 receptors (sIL-6Rs). Neurotoxic concentrations of PRPc-Aβ peptide complexes at dendrites, synaptogyrin-3- P-tau complexes at synapses, and IL-6-sIL-6R complexes accumulate both in and on neurons of origin at the site of mTBI and in NDEs that transport them into neighboring neurons not damaged directly by the inciting mTBI (Figure 1). Sustained increases in aquaporins promote edema and in IL-6 maintain chronic inflammation after mTBIs. Thus acute changes of mTBI may progress to pathogenesis of early CTE with increasing probability after subsequent repetitive mTBIs (Figure 2).

The postulated stages of mTBI induction of CTE are illustrated schematically from normal to mild deterioration after one mTBI to advanced CTE pathology after repeated mTBIs (mTBIn) (Figure 2). After a single mTBI, APP and BACE1 in exosomes from reactive astrocytes (AG) and to a lesser extent from damaged neurons are transferred to lesional and distant neurons. This initiates increased neuronal production of Aβ peptides, their oligomerization to Aβo that is catalyzed in part by increased levels of XIIIa (Hur et al., 2019), and beginning aggregation into loose deposits on neurons (Figure 2, second stage). IL-6-sIL-6R complexes in exosomes from reactive AG and microglia (MG) are secreted in part in their respective ADEs and MDEs for transfer to neurons, which mediates a sustained neuronal inflammatory response. PRPc-Fyn kinase-Aβ peptide complexes with XIIIa-oligomerized Aβ (Aβo) are transferred by NDEs to dendrites of other neurons and synaptogyrin-3 (SNGY-3)-P-tau complexes are transferred by NDEs and concentrated in axonal and presynaptic areas of other neurons to begin formation of loose P-tau strands and tangles. Repetitive mTBIs result in enhancement of all of these exosomal transfer processes with greater neuronal proteinopathic deposition of Aβo and forms of P-tau followed by early neuronal apoptosis in stage 3 (Figure 2). In summary, mTBI-derived CTE progression factors such as PRPc, XIIIa and SNGY-3, may act by binding to, concentrating and delivering to neurons neurotoxic Aβ peptides and various forms of P-tau. Others act independently of neurotoxic mediators, as exemplified by IL-6, which is bound by sIL-6Rs that dominate in neural tissues, link as the IL-6-sIL-6R complex to glycoprotein 130 on neurons and transmit neurodegenerative signals to the affected neurons (Erta et al., 2012; Rothaug et al., 2016). Accessory ligands for aquaporins have not been identified.

The many means for validation of the progression factor hypothesis of mediation of CTE by repetitive mTBIs include simple definitions of the time-courses of changes in NDE levels of three classes of relevant biomarkers. First, NDE levels of acute biomarkers, such as annexin VII, claudin-5, UCH-L1, spectrin fragments and NKCC-1, should only be elevated in the several weeks after each mTBI. Second, acute NDE increases in levels of Aβ peptide and P-tau mediators of proteinopathic toxicity should increase progressively and persist longer after each episode of mTBI. Third, NDE levels of the progression factors XIIIa, PRPc, SNGY-3, and possibly IL-6 and aquaporins should remain on elevated plateaus for months to years between episodes of mTBI. It also remains to be established if there are any relationships between the severity of a single mTBI or the stage of advancement to CTE and the NDE level of any of the biomarkers. Changes in the NDE level and duration of elevation of some biomarkers of mTBI also hopefully may provide information about the effectiveness of a therapeutic agent or program. Finally, this model may encourage extending drug discovery for mTBI and possibly Alzheimer disease to agents that alter neuronal expression of progression factors and their binding of Aβ peptides and P-tau species.

## Data Availability

The datasets for this manuscript are not publicly available because data are available in Goetzl et al. (2019) cited reference. Requests to access the datasets should be directed to edward.goetzl@ucsf.edu.

## Ethics Statement

This study was carried out in accordance with the recommendations of the University of California, San Francisco, San Francisco, CA, United States and University of Denver Human Research Institutional Review Committees with written informed consent from all subjects. All subjects gave written informed consent in accordance with the Declaration of Helsinki. The protocol was approved by the University of California, San Francisco and University of Denver Human Research Institutional Review Committees.

## Author Contributions

EG and DK formulated the conceptual framework of this perspective and prepared the draft manuscript. All authors analyzed the findings reported, edited the manuscript, and approved the final version submitted.

## Conflict of Interest Statement

The authors declare that the research was conducted in the absence of any commercial or financial relationships that could be construed as a potential conflict of interest. The handling Editor declared a past co-authorship with one of the authors DK.

## Abbreviations

Aβ42: β amyloid peptide 1-42
APP: amyloid precursor protein
BACE-1: beta-secretase-1 or beta-site amyloid precursor protein cleaving enzyme 1
BBB: blood-brain barrier
CTE: chronic traumatic encephalopathy
DU: University of Denver
MCP: monocyte chemoattractant protein
mTBI: mild TBI
NDE: neuron-derived exosome
NKCC-1: sodium-potassium-chloride co-transporter-1
PRPc: cellular prion protein
Rab 7: Rab 10, Rab 35, ras-related small GTPase 7, 10, 35
TBI: traumatic brain injury
UC: University of California
UCH-L1: ubiquitin C-terminal hydrolase L1
XIIIa: coagulation factor XIII.
